# Off-Hours Robotic-Assisted Spine Instrumentation in a Teaching Hospital: Feasibility Under Increased Emergency and Workflow Burden

**DOI:** 10.3390/jcm15155803

**Published:** 2026-07-24

**Authors:** Julien N. Jost, Kristina Catalano, Thomas Rhomberg, Debora Cipriani, Jaqueline Lattmann, Lukas Andereggen, Gerrit A. Schubert, Markus Bruder

**Affiliations:** 1 Department of Neurosurgery, Kantonsspital Aarau, 5001 Aarau, Switzerland; 2 Faculty of Medicine, University of Bern, 3012 Bern, Switzerland; 3 Department of Neurosurgery, RWTH Aachen University, 52062 Aachen, Germany; 4 Department of Neurosurgery, University Hospital Frankfurt, 60596 Frankfurt, Germany

**Keywords:** robotic-assisted spine surgery, off-hours surgery, teaching hospital, pedicle screw accuracy, emergency spine surgery, workflow burden

## Abstract

**Background/Objectives**: Robotic-assisted spine instrumentation is resource-intensive and may be challenging during off-hours emergency care. This study evaluated its operational feasibility and technical reliability in a teaching hospital. **Methods**: We performed an exploratory post hoc secondary analysis of 146 consecutive robotic-assisted spine instrumentation procedures comprising 1006 pedicle or sacral–alar–iliac screws. Off-hours surgery was defined as night and/or weekend surgery. The primary outcome was screw-level Gertzbein–Robbins (GR) accuracy. Secondary outcomes included workflow, perioperative, and 30-day clinical measures. Because all off-hours procedures were emergencies, an additional sensitivity analysis compared off-hours with regular-hours emergency procedures. **Results**: Sixteen procedures comprising 150 screws were performed off-hours. In the unadjusted primary comparison, off-hours cases had greater trauma and emergency burden, more frequent repeat imaging or re-registration, higher intensive care unit (ICU) utilization, and longer hospitalization. GR A/B accuracy was 96.0% off-hours and 98.2% during regular hours, while GR C–E rates were 4.0% and 1.8%, respectively. Severe deviations were uncommon, and no screw-related postoperative neurological deficit occurred. In the emergency-only analysis, GR A/B accuracy was 96.0% off-hours and 96.7% during regular-hours emergency surgery, while several perioperative differences were attenuated. **Conclusions**: Robotic-assisted spine instrumentation remained operationally feasible during off-hours emergency surgery, with high screw accuracy and few severe deviations. However, the small and highly selected off-hours subgroup and marked case-mix differences preclude conclusions regarding equivalence, comparative safety, or an independent effect of surgical timing. These findings should be considered hypothesis-generating.

## 1. Introduction

Spinal instrumentation performed during nights and weekends represents a distinct clinical and organizational scenario. Compared with scheduled elective practice, off-hours procedures are often driven by unstable trauma, progressive neurological compromise, infection, tumor-related instability, or other urgent indications. These cases may be exposed to multiple sources of operational stress, including time pressure, potentially reduced staffing, variable availability of subspecialized personnel, fatigue, and increased logistical complexity. In surgical care more broadly, the so-called weekend or after-hours effect has been associated with adverse outcomes in several administrative and population-based studies, although the magnitude and causality of this effect remain debated and are strongly influenced by case mix, institutional structure, and resource availability [1,2,3].

Spine surgery is particularly sensitive to these conditions because technical precision is required even when clinical acuity is high. Pedicle screw placement remains a cornerstone of posterior spinal stabilization [4,5]. Screw malposition can lead to neurological, vascular, visceral, or biomechanical complications, and even minor deviations may trigger intraoperative rescue steps, additional imaging, or postoperative surveillance. The Gertzbein–Robbins (GR) classification is widely used to grade pedicle screw accuracy and to distinguish clinically acceptable screw placement from potentially relevant cortical breaches [6].

Image-guided navigation and intraoperative three-dimensional (3D) imaging have been introduced to improve the accuracy and verification of pedicle screw placement compared with conventional fluoroscopic techniques [7,8,9,10,11,12]. Robotic-assisted spine instrumentation has been introduced to improve workflow reproducibility and screw placement accuracy. Previous work from our institution has described the integration of the CIRQ Robotic Alignment Module and the subsequent implementation of Loop-X intraoperative cone-beam computed tomography (CBCT) within this established robotic-assisted pedicle screw program [13,14,15]. However, robotic instrumentation is not a low-resource intervention. Its use requires a robotic guidance platform, intraoperative 3D imaging, navigation infrastructure, dedicated instrument trays, troubleshooting, and operating room (OR) personnel familiar with the workflow [14,15,16]. These requirements may become particularly challenging during nights and weekends, when specialized spine OR teams may not be consistently available.

This issue may be particularly relevant in teaching hospitals, where emergency spine procedures are performed within supervised training structures and where surgeon and operating-room team experience may vary according to timing, case complexity, and personnel availability. Off-hours robotic-assisted instrumentation may therefore combine high clinical acuity with potentially variable access to specialized resources and heterogeneous workflow experience. Most of the available robotic spine literature focuses on screw accuracy, radiation exposure, workflow, or learning curves in elective or mixed cohorts [16,17,18,19,20]. More broadly, the clinical evidence supporting emerging robotic applications in spine surgery remains heterogeneous and is often based on small observational cohorts or highly selected indications. Recent reviews have therefore emphasized the need for further investigation of implementation, learning curves, comparative effectiveness, and cost-effectiveness across different robotic platforms [21]. In contrast, the feasibility of maintaining a resource-intensive robotic workflow during off-hours emergency service remains insufficiently described. This represents a relevant implementation gap, particularly for teaching hospitals that must provide emergency spine care outside regular OR conditions.

The aim of this study was therefore not to demonstrate superiority of robotic-assisted instrumentation over conventional techniques, but to evaluate whether a structured robotic workflow remains feasible and technically reliable in high-acuity off-hours spine surgery. We hypothesized that off-hours cases would represent a more complex emergency subgroup with greater logistical and clinical burden, but that robotic-assisted screw placement would maintain high technical accuracy.

## 2. Materials and Methods

### 2.1. Study Design and Setting

This retrospective single-center study was an exploratory post hoc secondary analysis of a consecutive cohort of robotic-assisted spine instrumentation procedures performed at a tertiary teaching hospital between 25 May 2023 and 15 January 2026. The timing-specific research question and analyses were developed after establishment of the underlying cohort and were not based on a prospectively registered statistical analysis plan. The underlying cohort has previously been analyzed with respect to axial screw trajectory modulation, imaging implementation, and technical performance. The present study addresses a distinct research question focused on off-hours feasibility, emergency case mix, workflow burden, and technical reliability. Previously reported learning-curve analyses were not repeated [13,14,15]. The cohort included the full clinical spectrum of elective, urgent, and emergency robotic-assisted instrumentation procedures.

### 2.2. Teaching Hospital Context and Surgeon Experience

The department operates within a supervised teaching-hospital structure. Senior surgeons were defined as board-certified neurosurgeons with formal spine subspecialty certification, whereas junior surgeons were board-certified neurosurgeons without formal spine subspecialty certification at the time of surgery. This distinction reflects additional spine-specific training and certification rather than general neurosurgical board status. Junior surgeons performed procedures within the institutional supervision framework, with senior spine surgeons available for intraoperative guidance and intervention when required.

### 2.3. Patient Cohort and Exposure Definition

All included procedures involved robotic-assisted pedicle screw or second sacral alar-iliac (S2AI) screw placement. The primary exposure was off-hours surgery, defined according to incision time as procedures performed during night hours and/or weekends. Night hours were defined as procedures with incision time between 5:00 P.M. and 7:00 A.M. on any day. Weekend daytime surgery was defined as procedures with incision time on Saturday or Sunday between 7:00 A.M. and 5:00 P.M. Weekend night cases were captured by the night-hours definition and counted once as off-hours procedures. Regular-hours cases comprised procedures with incision time on weekdays between 7:00 A.M. and 5:00 P.M. Because the objective was to evaluate real-world off-hours feasibility, off-hours cases were not matched or restricted to elective indications. Trauma status, emergency status, surgeon experience, imaging modality, surgical approach, anatomical region, and number of screws were recorded for descriptive analyses. The regular-hours cohort included both elective and emergency procedures, whereas all procedures classified as off-hours were emergency operations. Consequently, surgical timing was strongly associated with urgency and case mix and was not interpreted as an isolated causal exposure. Baseline clinical variables included age, sex, body mass index (BMI), American Society of Anesthesiologists (ASA) physical status, cardiovascular disease, diabetes mellitus, anticoagulant therapy, and polytrauma status. These variables were evaluated descriptively to characterize potential differences in comorbidity and injury burden.

### 2.4. Robotic Workflow and Imaging

All procedures were performed using the CIRQ Robotic Alignment Module integrated with Brainlab navigation (Brainlab AG, Munich, Germany) [14,15,22,23]. Intraoperative imaging was performed using either 3D C-arm imaging with the Ziehm Vision RFD 3D system (Ziehm Imaging, Nuremberg, Germany) or Loop-X intraoperative CBCT (Brainlab AG, Munich, Germany), according to availability and workflow phase. The workflow required access to dedicated robotic instrument trays and personnel capable of performing robotic setup, sterile draping, intraoperative image acquisition, registration, and troubleshooting. The composition and prior robotic experience of the operating-room team were not systematically recorded and may have varied between procedures. Navigation was performed using intraoperative 3D image datasets acquired with either the Ziehm 3D C-arm or Loop-X CBCT, followed by registration to the Brainlab navigation environment and robotic trajectory alignment using the CIRQ module.

The core technical workflow was applied according to the same institutional principles during regular-hours and off-hours procedures. The robotic alignment platform, navigation environment, intraoperative three-dimensional imaging workflow, registration process, and trajectory-verification strategy were not intentionally modified according to surgical timing. Off-hours procedures were performed within the institution’s routine emergency and on-call operating-room structure rather than under a separate robotic protocol.

Detailed information regarding operating-room staffing levels, the individual robotic experience of scrub and circulating personnel, availability of dedicated spine operating-room staff, fatigue, anesthesia-related workflow, and logistical delays was not prospectively or systematically recorded. Accordingly, the present study cannot quantify staffing-related differences between regular-hours and off-hours procedures or determine their contribution to technical workflow outcomes.

Repeat scan or re-registration was documented only when additional intraoperative imaging or registration steps were required because of uncertainty regarding navigation accuracy, registration quality, patient movement, complex fracture anatomy, or other technical concerns. Routine final control scans performed as part of the standard robotic workflow were not counted as repeat scans. This endpoint was therefore interpreted as an intraoperative workflow rescue or safety-verification step rather than as a complication.

### 2.5. Outcomes

The primary outcome was screw-level accuracy using the GR classification [6]. Screws graded A or B were considered acceptable; screws graded C, D, or E were classified as relevant deviations. Severe deviations were defined as GR D or E. Case-level accuracy was additionally assessed as the occurrence of at least one GR C–E screw within a procedure.

Secondary outcomes included postoperative screw repositioning, repeat scan or re-registration, operative time, estimated blood loss (EBL), blood loss >1000 mL, length of stay (LOS), intensive care unit (ICU) admission, rehabilitation discharge, 30-day complications, Clavien-Dindo grade III or higher complications, 30-day readmission, 30-day reoperation, and postoperative screw-related neurological deficits [24].

Pre-incision preparation time was additionally evaluated as an available workflow metric. This variable represented the documented interval from positioning and radiographic level localization to skin incision. Component-specific timestamps for anesthesia clearance, robotic setup, image acquisition, registration, and repeat imaging or re-registration were not separately available.

### 2.6. Statistical Analysis

Continuous variables are reported as median with interquartile range and were compared using the Wilcoxon rank-sum test. Categorical variables are reported as counts and percentages and were compared using Fisher exact tests. Screw-level GR C–E rates were compared descriptively and by Fisher exact testing. Because screws were clustered within surgical cases and the number of malposition events was low, screw-level comparisons were interpreted descriptively. Case-level accuracy was therefore also reported as the presence of at least one GR C–E screw per procedure. Univariable logistic regression models estimated odds ratios for key outcomes comparing off-hours with regular-hours cases. Because the off-hours group was small and several outcomes were rare, regression analyses were interpreted as exploratory rather than confirmatory. All analyses were exploratory and hypothesis-generating. The study was not designed to estimate an independent causal effect of surgical timing. Because all off-hours procedures were emergency operations, an additional post hoc sensitivity analysis was performed after restricting the cohort to emergency procedures. This analysis compared off-hours emergency procedures with emergency procedures performed during regular hours and was intended to partially reduce confounding by urgency and the marked elective–emergency imbalance of the primary comparison.

No formal adjustment for multiple comparisons was applied because the study was not designed for confirmatory testing of multiple independent hypotheses. Accordingly, *p* values are reported as descriptive measures of statistical compatibility and should not be interpreted in isolation as definitive evidence for or against an association. Interpretation focused on effect estimates, confidence intervals, absolute event rates, clinical relevance, and the overall consistency of observed patterns.

Multivariable regression and propensity-based analyses were considered but were not performed because only 16 off-hours procedures were available, several outcomes had few or no events, and no elective procedures were performed during off-hours. These characteristics resulted in limited covariate overlap and a substantial risk of model overfitting, unstable effect estimates, and model-dependent extrapolation. The emergency-only analysis was therefore interpreted descriptively and exploratorily and was not considered an adjusted estimate of the causal effect of surgical timing.

Outcomes with zero events in one group were reported descriptively and not emphasized in the main odds-ratio forest plot. Statistical analyses were performed using R statistical software, version 4.5.2 (R Foundation for Statistical Computing, Vienna, Austria).

## 3. Results

### 3.1. Cohort Composition and Case Mix

Baseline, surgical, and perioperative characteristics are summarized in Table 1. The cohort comprised 146 robotic-assisted spine instrumentation procedures with 1006 screws. Sixteen procedures and 150 screws were performed off-hours, whereas 130 procedures and 856 screws were performed during regular hours. Off-hours cases represented a distinctly higher-acuity subgroup, with a higher proportion of trauma (87.5% vs. 17.7%) and emergency surgery (100% vs. 19.2%). All 16 off-hours procedures were emergency operations, whereas the regular-hours cohort included 25 emergency and 105 non-emergency procedures. The primary timing comparison therefore also represented a comparison between markedly different urgency and case-mix distributions. Measured baseline comorbidities did not show clear differences between the timing groups. Median ASA physical status was 3 [2–3] in both groups. Cardiovascular disease was present in 11/16 off-hours procedures (68.8%) and 77/130 regular-hours procedures (59.2%), diabetes mellitus in 4/16 (25.0%) and 28/130 (21.5%), and anticoagulant therapy in 4/16 (25.0%) and 45/130 (34.6%), respectively. By contrast, polytrauma was substantially more frequent among off-hours procedures (43.8% vs. 6.9%), indicating greater injury burden within the off-hours cohort.

### 3.2. Screw Accuracy

**Figure 1:**
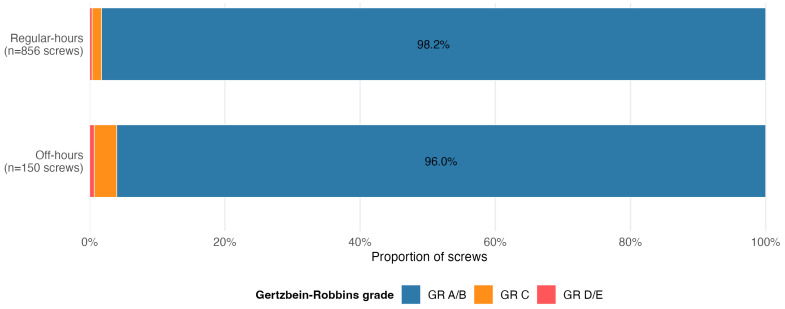
Screw-grade distribution by surgical timing. Stacked screw-level distribution of Gertzbein–Robbins (GR) grades comparing regular-hours and off-hours robotic-assisted instrumentation. Acceptable screw placement (GR A/B) remained high in both groups, whereas relevant GR C–E and severe GR D/E deviations were uncommon.

Screw-level distribution and accuracy are summarized in Table 2 and Figure 1. Off-hours instrumentation was predominantly thoracic (108/150 screws, 72.0%), whereas regular-hours instrumentation more often involved the lumbosacral spine (503/856 screws, 58.8%). Overall, 985 of 1006 screws were graded GR A/B, corresponding to an acceptable screw placement rate of 97.9%. Regular-hours cases had 841 of 856 screws graded GR A/B (98.2%), whereas off-hours cases had 144 of 150 screws graded GR A/B (96.0%). Relevant GR C–E deviations occurred in 15 regular-hours screws and 6 off-hours screws, corresponding to screw-level GR C–E rates of 1.8% and 4.0%, respectively. Severe GR D/E deviations were rare in both groups.

### 3.3. Perioperative Burden

Off-hours cases showed higher perioperative and resource burden in the unadjusted primary comparison. Median estimated blood loss was 350 [280–1100] mL during off-hours and 250 [150–500] mL during regular hours, whereas median length of stay was 8.5 [6–14] and 6 [4–10] days, respectively. ICU admission occurred in 9/16 off-hours procedures (56.2%) and 14/130 regular-hours procedures (10.8%). These differences should not be attributed to surgical timing or emergency status alone. They likely reflect a combination of greater trauma and polytrauma burden, procedural extent, anatomical complexity, postoperative monitoring requirements, and other measured and unmeasured patient-related factors. Despite the more frequent use of repeat imaging or re-registration during off-hours procedures, total operating-room time was numerically shorter during off-hours than during regular hours (187 [135–245] vs. 233.5 [170–320.3] min; *p* = 0.145). Pre-incision preparation time was also shorter in the exploratory unadjusted comparison (14.5 [13.0–22.3] vs. 24.0 [16.0–33.8] min; *p* = 0.034). However, procedural composition differed substantially between groups. Instrumentation without interbody fusion was performed in 14/16 off-hours procedures (87.5%) compared with 37/130 regular-hours procedures (28.5%; *p* < 0.001). The regular-hours cohort therefore included a substantially larger proportion of procedures with additional interbody and reconstructive components.

### 3.4. Clinical and Technical Burden Beyond Screw Placement

Off-hours cases were also associated with increased workflow and clinical burden in descriptive and exploratory analyses. Repeat scan or re-registration occurred in 5 of 16 off-hours cases (31.2%) compared with 10 of 130 regular-hours cases (7.7%), indicating a greater need for intraoperative workflow rescue or verification. Postoperative screw repositioning was rare and occurred only in one regular-hours case involving three screws. ICU admission was more frequent off-hours (9/16, 56.2%) than during regular hours (14/130, 10.8%). By contrast, major short-term clinical endpoints such as 30-day complications and 30-day reoperation did not differ clearly between groups.

### 3.5. Univariable Associations

**Figure 2:**
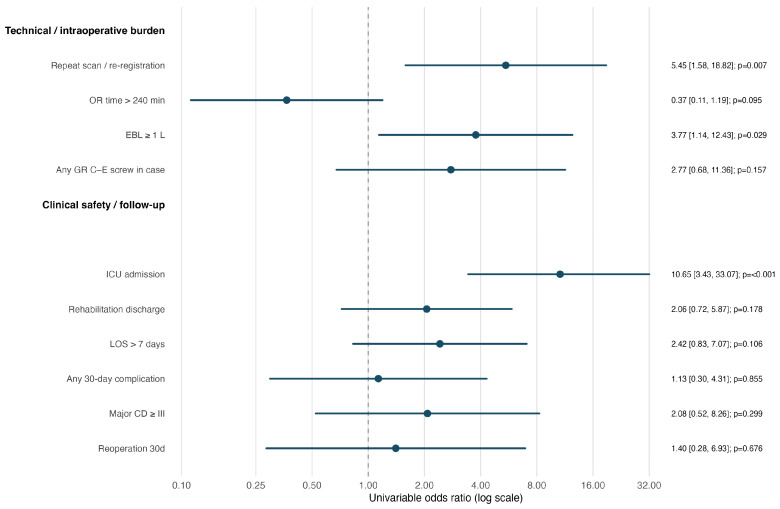
Exploratory unadjusted comparisons of off-hours and regular-hours procedures. Exploratory univariable odds ratios compare off-hours with regular-hours procedures and are presented with 95% confidence intervals. Estimates are unadjusted and should be interpreted descriptively because of the small off-hours subgroup, sparse outcome events, multiple comparisons, and marked differences in urgency and case mix. The dashed vertical line indicates an odds ratio of 1.0, representing no difference between off-hours and regular-hours procedures. OR, operating room; EBL, estimated blood loss; GR, Gertzbein–Robbins classification; ICU, intensive care unit; LOS, length of stay; CD, Clavien–Dindo; 30d, 30-day.

Univariable associations between off-hours timing and key outcomes are summarized in Figure 2. The pattern of associations suggests that differences were more apparent for technical and perioperative burden variables than for major short-term clinical endpoints. The exploratory unadjusted analyses showed higher technical and perioperative burden among off-hours cases, although these patterns were strongly influenced by differences in urgency and case mix.

### 3.6. Emergency-Only Sensitivity Analysis

Because all off-hours procedures were emergency operations, an exploratory post hoc sensitivity analysis was performed among the 41 emergency procedures, comprising 16 off-hours and 25 regular-hours cases. Restriction to emergency procedures substantially reduced several of the case-mix differences observed in the overall cohort. Trauma remained the predominant indication in both groups (87.5% vs. 92.0%; *p* = 0.637), and the number of implanted screws was similar (9 [8–10.5] vs. 8 [6–10]; *p* = 0.438).

Technical accuracy remained high in both emergency subgroups. At screw level, GR A/B accuracy was 96.0% during off-hours and 96.7% during regular-hours emergency surgery. At case level, at least one GR C–E screw was observed in 3/16 off-hours procedures (18.8%) and 5/25 regular-hours emergency procedures (20.0%; *p* = 1.000). Repeat scan or re-registration occurred in 5/16 (31.2%) and 5/25 (20.0%) procedures, respectively (*p* = 0.472).

Median operative time was 187 [135–245] min during off-hours and 215 [134–260] min during regular-hours emergency surgery (*p* = 0.593). Median estimated blood loss was 350 [280–1100] mL and 300 [150–700] mL (*p* = 0.243), whereas median length of stay was 8.5 [6–14] and 10 [7–12] days, respectively (*p* = 0.830). ICU admission remained numerically more frequent during off-hours emergency surgery (9/16, 56.2%) than during regular-hours emergency surgery (6/25, 24.0%; *p* = 0.051). Major complications classified as Clavien–Dindo grade III or higher occurred in 3/16 (18.8%) and 4/25 (16.0%) procedures, respectively (*p* = 1.000).

The distribution of primary surgeon experience was also comparable after restriction to emergency procedures. Junior surgeons were designated as primary surgeons in 10/16 off-hours procedures (62.5%) and 15/25 regular-hours emergency procedures (60.0%; *p* = 1.000). A senior surgeon was directly scrubbed in 10/16 (62.5%) and 17/25 (68.0%) procedures, respectively (*p* = 0.747).

Overall, restriction to emergency procedures attenuated several differences observed in the primary comparison. However, the small subgroup sizes, residual differences in emergency case characteristics, and limited number of outcome events preclude conclusions regarding equivalence or an independent effect of surgical timing. These findings are summarized in Table 3.

## 4. Discussion

The principal finding of this study is that off-hours robotic-assisted spine instrumentation was feasible in a real-world teaching hospital environment despite substantially increased clinical and logistical burden. Off-hours cases were dominated by emergency trauma surgery and were associated with greater perioperative burden, including longer hospitalization, higher ICU utilization, more frequent workflow rescue steps, and a tendency toward higher blood loss. These differences confirm that off-hours robotic cases were not simply elective cases performed at a different time of day, but a distinctly more complex subset of patients and procedures.

The emergency-only sensitivity analysis provides important context for the primary comparison. After restricting the cohort to emergency procedures, several differences in procedural and perioperative characteristics were attenuated, while technical accuracy remained high in both timing groups. This finding suggests that a substantial proportion of the differences observed in the overall cohort may reflect the marked elective–emergency case-mix imbalance rather than surgical timing alone. However, emergency procedures performed during regular hours and off-hours may still differ with respect to trauma severity, physiological condition, anatomical complexity, anticoagulant exposure, operative requirements, and postoperative monitoring needs. Residual confounding therefore remains likely, and the sensitivity analysis should not be interpreted as demonstrating equivalence or comparable safety.

The higher estimated blood loss, longer hospitalization, and more frequent ICU admission observed in the primary comparison are likely multifactorial. Although off-hours procedures were strongly enriched for emergency trauma and polytrauma, measured medical comorbidities and anticoagulant use did not show clear adverse imbalances in the off-hours group. Nevertheless, the available variables provide only a limited representation of baseline risk and clinical severity. Detailed information regarding trauma severity, hemodynamic instability, neurological urgency, preoperative hemoglobin and coagulation status, anticoagulant type and timing, reversal strategies, and the specific indication for postoperative ICU monitoring was not incorporated into the present analysis.

Procedural factors may also have influenced these outcomes. Estimated blood loss depends on the number of instrumented levels, surgical exposure, decompression, fracture morphology, associated injuries, interbody reconstruction, and intraoperative complexity. Similarly, ICU admission may reflect planned monitoring after major trauma, associated systemic injuries, physiological condition, or the timing of postoperative care rather than a complication of the robotic workflow. Length of stay is additionally influenced by postoperative recovery, rehabilitation requirements, discharge destination, social factors, and institutional care pathways. These outcomes should therefore be interpreted as markers of overall perioperative burden rather than as direct consequences of off-hours timing or robotic instrumentation.

The screw-grade distribution provides the central technical finding of this study. Despite the substantially higher emergency and trauma burden of off-hours cases, the proportion of acceptable GR A/B screws remained high (Figure 1). Relevant GR C–E deviations were uncommon, and severe GR D/E deviations were rare in both groups. Together with the observed increase in perioperative and workflow burden, these findings support the feasibility of maintaining a robotic-assisted instrumentation workflow under clinically and logistically adverse off-hours conditions.

The present findings address operational feasibility rather than comparative clinical or economic value. Although robotic-assisted instrumentation remained technically deployable during off-hours emergency surgery, the absence of a freehand or navigation-only control group precludes conclusions regarding whether robotic assistance provides superior outcomes or sufficient incremental benefit to justify its additional technological requirements. Conventional instrumentation may potentially achieve comparable outcomes with lower technology-related costs, and this question cannot be addressed by the present study. An important unresolved question is whether the technological and organizational costs of robotic-assisted instrumentation are offset by downstream clinical or economic benefits. Robotic workflows require capital investment, maintenance, dedicated instrumentation, intraoperative imaging, personnel training, and ongoing technical support. Some economic analyses have suggested that reductions in revision surgery, infection, operative time, or length of stay may partially offset these costs, whereas other comparative studies have reported higher index-procedure costs for robotic-assisted surgery [25,26,27]. Consequently, the economic value of robotic spine surgery is likely to depend on institutional case volume, platform-specific costs, procedural efficiency, case complexity, and the extent to which clinically consequential complications or revisions are avoided.

In the present cohort, postoperative screw repositioning was rare and no screw-related neurological deficit occurred. However, without a non-robotic comparator and direct cost data, these observations cannot be translated into attributable cost savings. Future comparative studies should prospectively capture capital and maintenance costs, consumables, staffing requirements, robotic setup and registration times, additional imaging, length of stay, intensive care utilization, readmission, unplanned revision surgery, and longer-term patient outcomes. Such analyses are required to determine whether maintaining robotic capability during off-hours emergency care provides incremental clinical or economic value beyond technical feasibility.

This point is particularly relevant because robotic-assisted spine instrumentation is a resource-intensive workflow. It requires a robotic arm, intraoperative imaging, navigation infrastructure, dedicated instrument sets, and personnel familiar with robotic setup, image acquisition, registration, and troubleshooting. These requirements may be more difficult to maintain during nights and weekends because staffing models, personnel experience, equipment access, and logistical support may differ from routine elective operating-room conditions. The extent to which these factors vary is institution-dependent and has rarely been characterized in studies of robotic-assisted emergency spine surgery.

The increased rate of repeat scan or re-registration deserves specific interpretation. In robotic and navigation-based surgery, additional imaging or re-registration may be perceived as a technical burden; however, it may also represent a safety mechanism. When registration quality, patient movement, anatomical complexity, or navigation confidence is uncertain, repeat imaging provides a structured opportunity for verification and correction. This interpretation is supported by prior studies showing that intraoperative 3D or CBCT may identify screw malposition and enable intraoperative correction before clinically relevant postoperative sequelae occur [28,29]. In this sense, workflow rescue should not be viewed simply as a complication, but rather as part of a safety-oriented verification strategy that may help preserve technical reliability in complex off-hours cases.

The numerically shorter total operating-room time during off-hours procedures should not be interpreted as evidence of greater robotic workflow efficiency. Total operating-room duration reflects the complete surgical procedure rather than the robotic component alone and was substantially influenced by procedural composition. Off-hours procedures predominantly consisted of emergency posterior stabilizations without interbody reconstruction, whereas the regular-hours cohort included a broader elective reconstructive spectrum with a substantially higher frequency of interbody procedures. This difference may have outweighed the additional time associated with repeat imaging or re-registration. Consistent with this interpretation, operative times were more similar after restriction to emergency procedures.

Surgeon experience represents another potential source of confounding. In the overall cohort, junior surgeons were more frequently designated as primary surgeons during off-hours procedures, although the difference was not statistically significant. However, this imbalance was no longer apparent after restriction to emergency procedures, in which the proportions of junior primary surgeons and directly scrubbed senior surgeons were similar between timing groups. This suggests that the initial difference may partly reflect emergency case allocation rather than surgical timing alone.

Nevertheless, the recorded classification of primary surgeon experience provides only a simplified representation of the teaching-hospital workflow. It does not quantify the degree of senior intraoperative guidance, task sharing, direct intervention, individual experience with the robotic platform, or the experience of the operating-room team. Furthermore, perioperative outcomes such as blood loss, ICU admission, and length of stay are strongly influenced by patient condition, trauma severity, anatomical complexity, and the extent of surgery. The present data therefore do not permit attribution of these outcomes to surgeon seniority.

Pre-incision preparation time was not prolonged during off-hours procedures; however, this measure represented the combined interval from positioning and level localization to skin incision and did not permit separation of individual workflow components. In particular, robotic setup, image acquisition, registration, and repeat-imaging durations were not individually recorded. The present data therefore cannot determine the specific time contribution of robotic workflow rescue steps or establish whether off-hours robotic workflow was more or less efficient.

The outcome patterns shown in Figure 2 were more pronounced for workflow and perioperative burden than for selected short-term clinical endpoints. However, the small off-hours subgroup, sparse events, and wide confidence intervals preclude conclusions regarding comparable safety. Rather, these findings support interpreting off-hours timing as a marker of a high-acuity emergency subgroup while showing that technical screw accuracy remained high despite increased workflow burden.

Future studies should extend these findings through multicenter prospective designs with larger off-hours cohorts and appropriate emergency and non-robotic comparison groups. In addition to technical accuracy and short-term clinical outcomes, such studies should prospectively capture robotic setup time, registration time, staffing requirements, workflow interruptions, radiation exposure, and direct and downstream costs. The broader robotic spine literature remains heterogeneous across platforms and indications, and additional evidence regarding learning curves, comparative effectiveness, and cost-effectiveness is required before the clinical and economic value of individual robotic systems can be determined [21].

### Limitations

This study has several limitations. First, the retrospective single-center design and the small, markedly imbalanced off-hours subgroup limited statistical precision and increased the risk of both type I and type II errors. The analyses were exploratory, no adjustment for multiple comparisons was performed, and non-significant findings should not be interpreted as evidence of equivalence or comparable safety.

Second, off-hours timing was strongly associated with emergency status, trauma burden, anatomical distribution, and procedural characteristics. Although the emergency-only sensitivity analysis reduced some of these differences, residual confounding remains likely. The limited number of off-hours procedures and outcome events also precluded robust multivariable or propensity-based adjustment. Accordingly, no independent or causal effect of surgical timing can be inferred.

Third, no conventional control group or direct cost data were available. Detailed information on staffing, fatigue, individual team experience, and component-specific robotic workflow times was also not systematically recorded. In addition, available variables incompletely captured trauma severity, physiological condition, anticoagulation management, and indications for ICU admission. The findings therefore reflect the experience of a single teaching hospital with an established robotic program and may not be generalizable to other institutional settings.

## 5. Conclusions

In this exploratory single-center cohort, robotic-assisted spine instrumentation remained operationally feasible during off-hours emergency surgery despite increased clinical acuity and workflow burden. Screw accuracy remained high, severe malposition was uncommon, and no screw-related postoperative neurological deficit was observed. However, the small and highly selected off-hours subgroup, the marked imbalance in urgency and case mix, and the absence of a non-robotic control group preclude conclusions regarding equivalence, comparative safety, cost-effectiveness, or an independent effect of surgical timing. The findings should therefore be interpreted as hypothesis-generating and support further multicenter studies with larger off-hours cohorts, adjusted analyses, and appropriate conventional comparison groups.

## Figures and Tables

**Table 1:** Case-level baseline, surgical, and perioperative characteristics according to surgical timing.

Characteristic	Regular-h (n = 130)	Off-h (n = 16)	*p* Value
**Demographics**			
Age, years	70 [59–77]	75 [56–83.5]	0.377
Female sex	62/130 (47.7%)	4/16 (25.0%)	0.112
BMI, kg/m^2^	27 [24–31]	26 [23.8–28.5]	0.723
ASA score	3 [2–3]	3 [2–3]	0.572
Cardiovascular disease	77/130 (59.2%)	11/16 (68.8%)	0.592
Diabetes mellitus	28/130 (21.5%)	4/16 (25.0%)	0.752
Anticoagulant therapy	45/130 (34.6%)	4/16 (25.0%)	0.579
**Indication/pathology**			
Polytrauma	9/130 (6.9%)	7/16 (43.8%)	<0.001
Trauma	23/130 (17.7%)	14/16 (87.5%)	<0.001
Degenerative disease	83/130 (63.8%)	1/16 (6.2%)	<0.001
Tumor	12/130 (9.2%)	0/16 (0%)	0.362
Infection	2/130 (1.5%)	0/16 (0%)	1.000
Emergency surgery	25/130 (19.2%)	16/16 (100%)	<0.001
**Surgical workflow**			
Primary surgeon experience	Junior: 53 (40.8%); Senior: 77 (59.2%)	Junior: 10 (62.5%); Senior: 6 (37.5%)	0.114
Senior scrubbed	103/130 (79.2%)	10/16 (62.5%)	0.200
Approach	Open: 35 (26.9%); Percutaneous: 95 (73.1%)	Open: 5 (31.2%); Percutaneous: 11 (68.8%)	0.768
Navigation modality	3D C-arm: 68 (52.3%); Loop-X: 62 (47.7%)	3D C-arm: 8 (50.0%); Loop-X: 8 (50.0%)	1.000
Number of screws per case	5 [4–8]	9 [8–10.5]	<0.001
Total OR time, min	233.5 [170–320.2]	187 [135–245]	0.145
Repeat scan/re-registration	10/130 (7.7%)	5/16 (31.2%)	0.013
Instrumentation without interbody fusion	37/130 (28.5%)	14/16 (87.5%)	<0.001
Pre-incision preparation time, min	24 [16–33.8]	14.5 [13–22.3]	0.034
**Perioperative course**			
Estimated blood loss, ml	250 [150–500]	350 [280–1100]	0.072
Length of stay, days	6 [4–10]	8.5 [6–14]	0.023
ICU admission	14/130 (10.8%)	9/16 (56.2%)	<0.001
Discharge to rehabilitation	50/130 (38.5%)	9/16 (56.2%)	0.187
**30-day outcomes**			
Any 30-day complication	22/130 (16.9%)	3/16 (18.8%)	0.738
Clavien-Dindo ≥ III	13/130 (10.0%)	3/16 (18.8%)	0.386
30-day readmission	9/130 (6.9%)	0/16 (0%)	0.598
30-day reoperation	12/130 (9.2%)	2/16 (12.5%)	0.653
New postoperative neurologic deficit	13/130 (10.0%)	0/16 (0%)	0.362

Values are median [interquartile range] or n/N (%), unless otherwise indicated. Regular-h, regular hours; Off-h, off-hours; ASA, American Society of Anesthesiologists; BMI, body mass index; 3D, three-dimensional; OR, operating room; ICU, intensive care unit; EBL, estimated blood loss; LOS, length of stay. *p* values are from Wilcoxon rank-sum tests for continuous variables and Fisher exact tests for categorical variables, as appropriate. Pre-incision preparation time was defined as the interval from positioning and radiographic level localization to skin incision.

**Table 2:** Screw-level distribution, accuracy, and postoperative screw repositioning according to surgical timing.

Characteristic	Regular-h (856 Screws; 130 Cases)	Off-h (150 Screws; 16 Cases)	*p* Value
**Screw-level distribution**			
Screw region	Cervical: 54 (6.3%); Thoracic: 299 (34.9%); Lumbosacral: 503 (58.8%)	Cervical: 4 (2.7%); Thoracic: 108 (72.0%); Lumbosacral: 38 (25.3%)	<0.001
**Screw-level accuracy**			
GR A/B screws	841/856 (98.2%)	144/150 (96.0%)	0.111
GR C–E screws	15/856 (1.8%)	6/150 (4.0%)	0.111
GR D/E screws	3/856 (0.4%)	1/150 (0.7%)	0.476
Postoperative screw repositioning	3/856 (0.4%)	0/150 (0%)	1.000
**Case-level accuracy/repositioning**			
Any GR C–E screw in case	10/130 (7.7%)	3/16 (18.8%)	0.155
Any GR D/E screw in case	3/130 (2.3%)	1/16 (6.2%)	0.375
Any postoperative screw repositioning in case	1/130 (0.8%)	0/16 (0%)	1.000
Screw-related postoperative neurologic deficit	0/130 (0%)	0/16 (0%)	—

Values are n/N (%), unless otherwise indicated. Regular-h, regular hours; Off-h, off-hours; GR, Gertzbein–Robbins classification. Screw region, GR grade, and postoperative screw repositioning are reported at screw level unless otherwise indicated. Case-level variables are reported per procedure.

**Table 3:** Exploratory sensitivity analysis restricted to emergency procedures according to surgical timing.

Characteristic	Regular-h Emergency (n = 25)	Off-h Emergency (n = 16)	*p* Value
Trauma	23/25 (92.0%)	14/16 (87.5%)	0.637
Number of screws per case	8 [6–10]	9 [8–10.5]	0.438
Total OR time, min	215 [134–260]	187 [135–245]	0.593
Repeat scan/re-registration	5/25 (20.0%)	5/16 (31.2%)	0.472
Estimated blood loss, ml	300 [150–700]	350 [280–1100]	0.243
Length of stay, days	10 [7–12]	8.5 [6–14]	0.830
ICU admission	6/25 (24.0%)	9/16 (56.2%)	0.051
Any GR C–E screw per case	5/25 (20.0%)	3/16 (18.8%)	1.000
Clavien–Dindo ≥ III	4/25 (16.0%)	3/16 (18.8%)	1.000
30-day reoperation	3/25 (12.0%)	2/16 (12.5%)	1.000
New postoperative neurological deficit	1/25 (4.0%)	0/16 (0%)	1.000
Instrumentation without interbody fusion	19/25 (76.0%)	14/16 (87.5%)	0.448
Pre-incision preparation time, min	26 [15–35]	14.5 [13–22.3]	0.089
Junior primary surgeon	15/25 (60.0%)	10/16 (62.5%)	1.000
Senior surgeon scrubbed	17/25 (68.0%)	10/16 (62.5%)	0.747
Age, years	72 [56–77]	75 [56–83.5]	0.669
ASA score	3 [2–3]	3 [2–3]	0.954
Cardiovascular disease	13/25 (52.0%)	11/16 (68.8%)	0.344
Diabetes mellitus	5/25 (20.0%)	4/16 (25.0%)	0.717
Anticoagulant therapy	9/25 (36.0%)	4/16 (25.0%)	0.513
Polytrauma	9/25 (36.0%)	7/16 (43.8%)	0.746

Values are median [interquartile range] or n/N (%). Regular-h, regular hours; Off-h, off-hours; ASA, American Society of Anesthesiologists; GR, Gertzbein–Robbins classification; OR, operating room; ICU, intensive care unit. *p* values are derived from Wilcoxon rank-sum tests for continuous variables and Fisher’s exact tests for categorical variables. The analysis was exploratory and was not adjusted for multiple comparisons.

## Data Availability

The datasets generated and analyzed during the current study are not publicly available due to institutional data protection regulations but are available from the corresponding author upon reasonable request and with approval of the local ethics committee.

## Abbreviations

The following abbreviations are used in this manuscript:

3D	three-dimensional
ASA	American Society of Anesthesiologists
BMI	body mass index
CBCT	cone-beam computed tomography
CIRQ	robotic alignment module
EBL	estimated blood loss
GR	Gertzbein–Robbins classification
ICU	intensive care unit
LOS	length of stay
OR	operating room
S2AI	second sacral alar-iliac screw

## References

[1] Aylin P., Alexandrescu R., Jen M.H., Mayer E.K., Bottle A. (2013). Day of Week of Procedure and 30 Day Mortality for Elective Surgery: Retrospective Analysis of Hospital Episode Statistics. BMJ.

[2] McIsaac D.I., Bryson G.L., van Walraven C. (2014). Elective, Major Noncardiac Surgery on the Weekend: A Population-Based Cohort Study of 30-Day Mortality. Med. Care.

[3] Glance L.G., Osler T., Li Y., Lustik S.J., Eaton M.P., Dutton R.P., Dick A.W. (2016). Outcomes Are Worse in US Patients Undergoing Surgery on Weekends Compared with Weekdays. Med. Care.

[4] Gaines R.W. (2000). The Use of Pedicle-Screw Internal Fixation for the Operative Treatment of Spinal Disorders. J. Bone Jt. Surg. Am..

[5] Zindrick M.R., Wiltse L.L., Doornik A., Widell E.H., Knight G.W., Patwardhan A.G., Thomas J.C., Rothman S.L., Fields B.T. (1987). Analysis of the Morphometric Characteristics of the Thoracic and Lumbar Pedicles. Spine.

[6] Gertzbein S.D., Robbins S.E. (1990). Accuracy of Pedicular Screw Placement In Vivo. Spine.

[7] Tian N.-F., Huang Q.-S., Zhou P., Zhou Y., Wu R.-K., Lou Y., Xu H.-Z. (2011). Pedicle Screw Insertion Accuracy with Different Assisted Methods: A Systematic Review and Meta-Analysis of Comparative Studies. Eur. Spine J..

[8] Gelalis I.D., Paschos N.K., Pakos E.E., Politis A.N., Arnaoutoglou C.M., Karageorgos A.C., Ploumis A., Xenakis T.A. (2012). Accuracy of Pedicle Screw Placement: A Systematic Review of Prospective in Vivo Studies Comparing Free Hand, Fluoroscopy Guidance and Navigation Techniques. Eur. Spine J..

[9] Mason A., Paulsen R., Babuska J.M., Rajpal S., Burneikiene S., Nelson E.L., Villavicencio A.T. (2014). The Accuracy of Pedicle Screw Placement Using Intraoperative Image Guidance Systems: A Systematic Review. J. Neurosurg. Spine.

[10] Tabaraee E., Gibson A.G., Karahalios D.G., Potts E.A., Mobasser J.-P., Burch S. (2013). Intraoperative Cone Beam–Computed Tomography With Navigation (O-ARM) Versus Conventional Fluoroscopy (C-ARM): A Cadaveric Study Comparing Accuracy, Efficiency, and Safety for Spinal Instrumentation. Spine.

[11] Cordemans V., Kaminski L., Banse X., Francq B.G., Detrembleur C., Cartiaux O. (2017). Pedicle Screw Insertion Accuracy in Terms of Breach and Reposition Using a New Intraoperative Cone Beam Computed Tomography Imaging Technique and Evaluation of the Factors Associated with These Parameters of Accuracy: A Series of 695 Screws. Eur. Spine J..

[12] Ohashi H., Kawamura D., Hatano K., Ohashi S., Tochigi S., Isoshima A., Nagashima H., Otani K., Karagiozov K., Tani S. (2023). Intraoperative Cone-Beam Computed Tomography Assessment of Spinal Pedicle Screws Placement Precision Is in Full Agreement with Postoperative Computed Tomography Assessment. World Neurosurg..

[13] Jost J.N., Catalano K., Lattmann J., Rhomberg T., Cipriani D., Schubert G.A., Andereggen L., Bruder M. (2026). Axial Convergence in Percutaneous Robotic Pedicle Screw Placement: Mixed-Effects Analysis of 1006 Screws. Spine Open.

[14] Jost J.N., Catalano K., Lattmann J., Cipriani D., Rhomberg T., Andereggen L., Schubert G.A., Bruder M. (2026). Implementation of Intraoperative Cone-Beam CT (Loop-X) in an Established Robotic-Assisted Pedicle Screw Program: An Epoch-Based Cohort Study. J. Clin. Med..

[15] Jost J., Cipriani D., Schubert G., Bruder M. (2024). Integration and Impact of a Robotic Guidance System in a Spinal Navigation Unit: A Single Center Retrospective Study of 333 Screws. Brain Spine.

[16] Gautam D., Vivekanandan S., Mazur M.D. (2024). Robotic Spine Surgery: Systematic Review of Common Error Types and Best Practices. Oper. Neurosurg..

[17] MacLean L., Hersh A.M., Bhimreddy M., Jiang K., Davidar A.D., Weber-Levine C., Alomari S., Judy B.F., Lubelski D., Theodore N. (2024). Comparison of Accuracy, Revision, and Perioperative Outcomes in Robot-Assisted Spine Surgeries: Systematic Review and Meta-Analysis. J. Neurosurg. Spine.

[18] Pennington Z., Judy B.F., Zakaria H.M., Lakomkin N., Mikula A.L., Elder B.D., Theodore N. (2022). Learning Curves in Robot-Assisted Spine Surgery: A Systematic Review and Proposal of Application to Residency Curricula. Neurosurg. Focus.

[19] Torii Y., Ueno J., Iinuma M., Yoshida A., Niki H., Akazawa T. (2022). The Learning Curve of Robotic-Assisted Pedicle Screw Placements Using the Cumulative Sum Analysis: A Study of the First 50 Cases at a Single Center. Spine Surg. Relat. Res..

[20] Lieberman I.H., Kisinde S., Hesselbacher S. (2020). Robotic-Assisted Pedicle Screw Placement During Spine Surgery. JBJS Essent. Surg. Tech..

[21] Choucha A., Travaglini F., De Simone M., Evin M., Farah K., Fuentes S. (2025). The Da Vinci Robot, a Promising yet Underused Minimally Invasive Tool for Spine Surgery: A Scoping Review of Its Current Role and Limits. Neurochirurgie.

[22] Gabrovsky N., Ilkov P., Laleva M. (2023). Cirq® Robotic Assistance for Thoracolumbar Pedicle Screw Placement—Feasibility, Accuracy, and Safety. Brain Spine.

[23] Gabrovsky N., Ilkov P., Laleva M. (2023). Cirq Robotic Assistance for Thoracolumbar Pedicle Screw Placement: Overcoming the Disadvantages of Minimally Invasive Spine Surgery. Acta Neurochir. Suppl..

[24] Clavien P.A., Barkun J., de Oliveira M.L., Vauthey J.N., Dindo D., Schulick R.D., de Santibañes E., Pekolj J., Slankamenac K., Bassi C. (2009). The Clavien-Dindo Classification of Surgical Complications: Five-Year Experience. Ann. Surg..

[25] Menger R.P., Savardekar A.R., Farokhi F., Sin A. (2018). A Cost-Effectiveness Analysis of the Integration of Robotic Spine Technology in Spine Surgery. Neurospine.

[26] Sturgill D., How J., Blajda T., Davis Z., Ali M., O’Malley G., Patel N.V., Khan M.F., Goldstein I. (2024). Are the Clinical Outcomes and Cost-Effectiveness of Robot-Assisted Pedicle Screw Placement in Lumbar Fusion Surgery Superior to Computed Tomography Navigation and Freehand Fluoroscopy-Guided Techniques? A Systematic Review and Network Meta-Analysis. World Neurosurg..

[27] Ezeokoli E.U., Pfennig M., John J., Gupta R., Khalil J.G., Park D.K. (2022). Index Surgery Cost of Fluoroscopic Freehand Versus Robotic-Assisted Pedicle Screw Placement in Lumbar Instrumentation: An Age, Sex, and Approach-Matched Cohort Comparison. J. Am. Acad. Orthop. Surg. Glob. Res. Rev..

[28] Zimmermann F., Kohl K., Privalov M., Franke J., Vetter S.Y. (2021). Intraoperative 3D Imaging with Cone-Beam Computed Tomography Leads to Revision of Pedicle Screws in Dorsal Instrumentation: A Retrospective Analysis. J. Orthop. Surg. Res..

[29] Baumgart L., Ille S., Kirschke J.S., Meyer B., Krieg S.M. (2023). Radiation Doses and Accuracy of Navigated Pedicle Screw Placement in Cervical and Thoracic Spine Surgery: A Comparison of Sliding Gantry CT and Mobile Cone-Beam CT in a Homogeneous Cohort. J. Neurosurg. Spine.

